# Adaptation and Implementation of the Dysphagia and Dysphonia Inventory (HSS-DDI) in Greek Patients After Anterior Surgical Removal of the Herniated Cervical Spine

**DOI:** 10.3390/diagnostics15161994

**Published:** 2025-08-09

**Authors:** Soultana Papadopoulou, Aliki I. Venetsanopoulou, Avraam Ploumis, Kalliopi Megari, Evaggelia-Maria Perivolioti, Nikoleta Tsipa, Andreas Zygouris, Spyridon Voulgaris

**Affiliations:** 1 Department of Speech Therapy, School of Health Sciences, University of Ioannina, 45500 Ioannina, Greece; evelinaperivolioti@gmail.com (E.-M.P.); nikoltsip@gmail.com (N.T.); 2 Laboratory of Molecular Immunology, Department of Biological Applications and Technology, School of Health Sciences, University of Ioannina, 45500 Ioannina, Greece; a.venetsanopoulou@uoi.gr; 3 Department of Physical Medicine and Rehabilitation, School of Medicine, University of Ioannina, 45500 Ioannina, Greece; aploumis@uoi.gr; 4 City College, University of York, Europe Campus, 54622 Thessaloniki, Greece; kmegari@psy.auth.gr; 5 Department of Neurosurgery, Faculty of Medicine, School of Health Sciences, University of Ioannina, 45500 Ioannina, Greece; an.zigouris@uoi.gr (A.Z.); svoulgar@uoi.gr (S.V.)

**Keywords:** anterior cervical discectomy and fusion, cervical spine herniation, postoperative dysphagia, postoperative dysphonia, risk factors, surgical outcomes, HSS-DDI

## Abstract

**Background**: Anterior cervical discectomy and fusion (ACDF) is a widely performed surgical intervention for cervical spine herniation (CSH) to alleviate symptoms such as pain, weakness, and restricted mobility. Despite its efficacy, ACDF is associated with postoperative complications, notably dysphagia and dysphonia (PDD). **Objective**: This study investigates the prevalence, severity, and risk factors associated with PDD following ACDF using the validated Dysphagia and Dysphonia Inventory (HSS-DDI) adapted into Greek. **Methods**: A prospective observational cohort study was conducted at the University General Hospital of Ioannina from May to November 2023. The study involved 40 adult patients who underwent ACDF for CSH. Postoperative dysphagia and dysphonia were assessed using the Ohkuma questionnaire and HSS-DDI at 1 week and 1 month postoperatively. **Results**: The mean age of participants was 54.78 years, with a majority being male (60%). In terms of body mass index (BMI), 30% of participants had a normal weight, 47.5% were overweight, and 22.5% were obese. This study revealed that dysphagia and dysphonia were common postoperative complications, with improvements noted after one month. Factors such as BMI were statistically significant in influencing dysphagia outcomes, with normal BMI individuals reporting better outcomes than obese participants. Confirmatory factor analysis indicated the need for a larger sample size to confirm subscale validity in the Greek population. **Conclusions**: Postoperative dysphagia and dysphonia are prevalent following ACDF, but most patients experience improvements within a short period. Identifying risk factors, such as BMI, and utilizing validated assessment tools like the HSS-DDI can help optimize surgical techniques and postoperative care. Further studies with larger sample sizes are recommended for a more comprehensive understanding of these complications.

## 1. Introduction

Anterior cervical discectomy and fusion (ACDF) is a widely performed surgical procedure for managing spine pathology, particularly in patients with cervical spine herniation (CSH). In the last twenty years, the use of ACDF has significantly increased, highlighting its significance in spinal surgery [1]. This procedure effectively alleviates symptoms such as pain, weakness, and restricted mobility caused by nerve root and spinal cord compression [2]. However, despite its efficacy, ACDF carries the risk of postoperative complications, with dysphagia and dysphonia (PDD) being the most frequently reported adverse effects [1,3].

Cervical spine herniation (CSH) occurs when the nucleus pulposus ruptures through the fibrous annulus, leading to compression of the meninges or spinal nerve roots [4]. This condition is a common cause of neck pain, particularly in middle-aged individuals, and can present with additional symptoms, such as radiculopathy, myelopathy, sensory disturbances, muscle weakness, restricted mobility, and, in severe cases, upper limb paresis [5]. The etiology of CSH is multifactorial, often resulting from traumatic injury or degenerative changes in the spine. In severe cases, surgical intervention via either an anterior or posterior approach is required [6]. ACDF is the preferred technique for relieving nerve root compression and improving the patient’s quality of life [3]. However, given the potential for postoperative complications, understanding the risks associated with ACDF is crucial for optimizing patient outcomes [1].

### 1.1. Postoperative Dysphagia and Dysphonia

Swallowing is a complex neuromuscular process that involves 25 pairs of muscles and five cranial nerves working in coordination with the respiratory cycle [7]. Dysphagia, or swallowing dysfunction, can impact any stage of swallowing, including the oral preparatory, oral transit, pharyngeal, and esophageal phases [8]. Postoperative dysphagia is a common complication of ACDF, with symptoms typically peaking around the second postoperative week before gradually improving [9]. Studies indicate that dysphagia occurs in approximately 50.2% of patients within the first month after surgery, 32.3% in the second month, 17.8% in the sixth month, and 12.5% after one year [10]. In 12% to 14% of patients, severe symptoms persist beyond one year [11], with permanent and severe dysphagia reported in 4% to 10% of patients [12].

Similarly, phonation, which involves air pressure passing through the vocal cords to produce sound [13], can be affected by ACDF. Dysphonia, defined as an alteration in vocal pitch, resonance, and quality, is another commonly reported postoperative complication [14].

Dysphonia after ACDF is often self-limiting, with most cases resolving within six months [15]. The exact mechanisms underlying postoperative dysphonia remain unclear but are thought to be associated with prolonged intubation, mechanical displacement of laryngeal structures, pharyngeal edema, or vocal cord paralysis [16]. Given its impact on communication and quality of life, early identification and management of dysphonia are essential.

### 1.2. Epidemiology and Risk Factors

**Figure 1:**
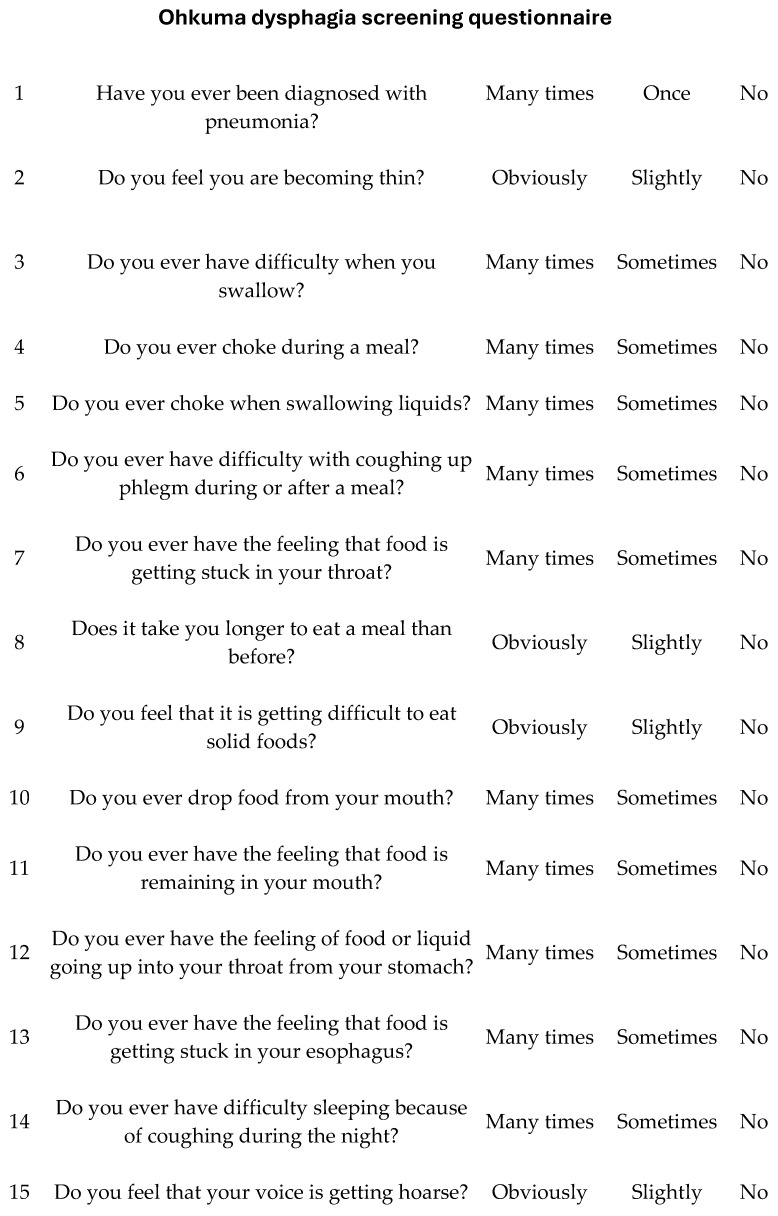
Contents of the Ohkuma dysphagia screening questionnaire.

**Figure 2:**
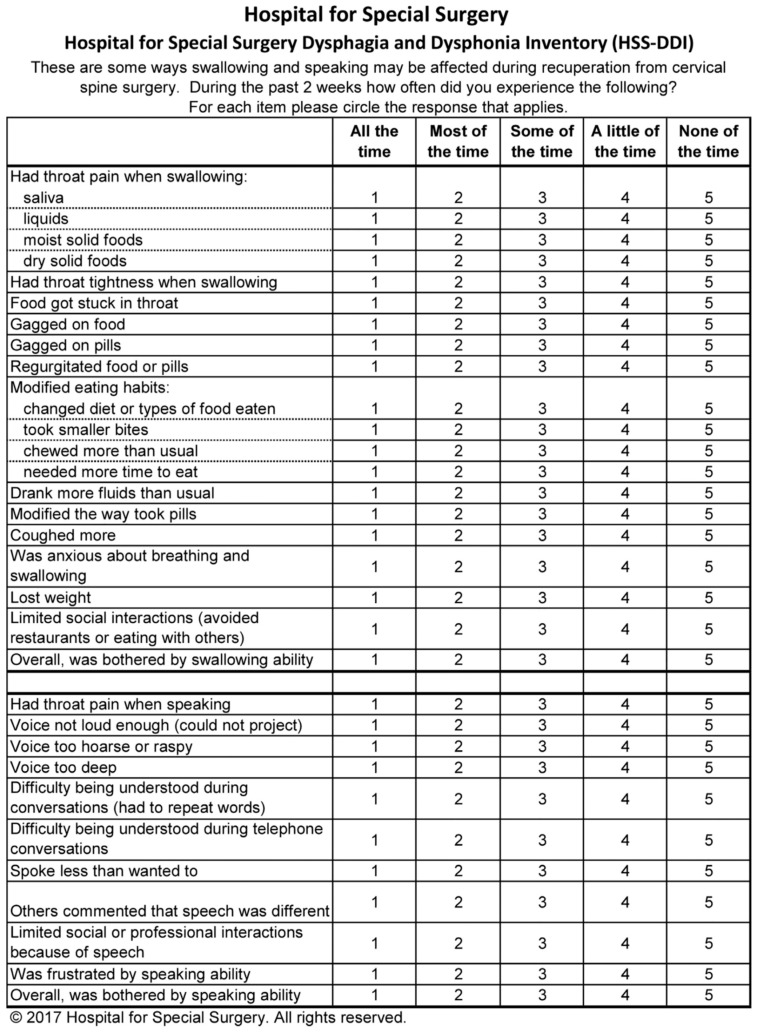
Hospital for Special Surgery Dysphagia and Dysphonia Inventory (HSS-DDI).

**Figure 3:**
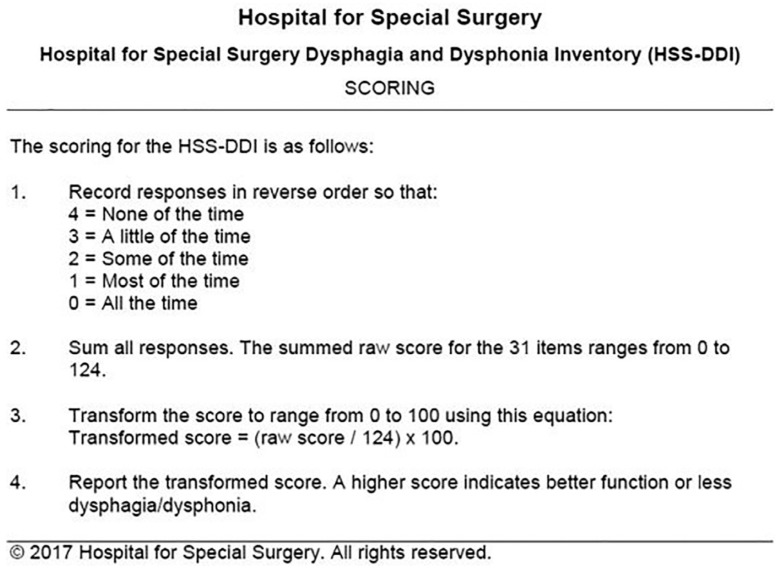
Scoring of the Hospital for Special Surgery Dysphagia and Dysphonia Inventory (HSS-DDI).

The reported prevalence of dysphagia and dysphonia following ACDF varies widely due to differences in assessment tools, surgical techniques, and definitions used in the literature. Dysphonia rates range from 3% to 67%, while dysphagia occurs in 1% to 51% of patients [17]. These complications are among the most common postoperative outcomes of ACDF, with some studies suggesting an incidence exceeding 80% [18]. Identifying risk factors for these complications is crucial for improving surgical outcomes. Several factors have been associated with an increased risk of PDD, including advanced age, female gender, smoking, preoperative opioid use, multilevel surgery (involving more than one vertebral level), and operations at higher cervical levels such as C3/C4 [2,10,11]. This study aims to investigate the prevalence, severity, and risk factors associated with PDD following ACDF. By employing the validated Ohkuma questionnaire (Figure 1), a standardized and internationally accepted tool for the detection of dysphagia, as well as the Dysphagia and Dysphonia Inventory (HSS-DDI)—a questionnaire specifically designed for post-ACDF patients that also assesses dysphonia (Figure 2 and Figure 3) and has been adapted into Greek—this study provides a comprehensive evaluation of these complications [19]. By documenting clinical characteristics and identifying contributing factors, this study will contribute to improving surgical techniques and postoperative care strategies for ACDF patients.

## 2. Materials and Methods

### 2.1. Research Questions

What is the prevalence and severity of postoperative dysphagia and dysphonia in Greek patients following anterior cervical discectomy and fusion?

How do dysphagia and dysphonia symptoms change over time (i.e., at 1 week and 4 weeks postoperatively)?

Which patient-related factors (e.g., BMI, age, and gender) are significantly associated with the severity and persistence of postoperative dysphagia and dysphonia?

How does the Greek version of the HSS-DDI compare to other assessment tools, such as the Ohkuma questionnaire, in terms of sensitivity and specificity for detecting postoperative complications?

### 2.2. Study Design and Participants

This prospective observational cohort study was conducted at the University General Hospital of Ioannina between May 2023 and November 2023. The study included patients who underwent anterior cervical discectomy and interbody fusion for cervical herniated disc removal. A total of 40 patients were enrolled, comprising 16 females and 24 males. Ethical approval for the study was obtained from the Ethics Council of the University of Ioannina (24 April 2023, 20766/2023). All participants provided written informed consent before inclusion in the study.

### 2.3. Inclusion and Exclusion Criteria

Eligible participants were adult patients (≥18 years) who had undergone anterior cervical discectomy for cervical herniated disc removal. Participants were required to demonstrate cognitive ability sufficient to complete study-related questionnaires and assessments independently and to express willingness to participate in the study.

Patients were excluded if they met any of the following criteria:

- Presence of a significant cognitive impairment preventing independent completion of questionnaires;
- Failure to provide informed consent;
- History of psychiatric illness;
- Undergoing posterior cervical discectomy instead of anterior cervical discectomy.

### 2.4. Patient Recruitment

Patients were recruited from three different sources:

1. University General Hospital of Ioannina (n = 9);
2. Physiotherapy centers (n = 18);
3. Rehabilitation centers collaborating with the study team (n = 13).

### 2.5. Data Collection and Clinical Assessments

Patient demographics, medical history, surgical details, and postoperative complications were recorded. Demographic data, including gender, age, occupation, marital status, medication use, history of psychiatric illness, and symptom onset, were collected using a standardized form. Clinical assessments were performed at multiple postoperative time points: 1 week and 1 month. Postoperative complications, specifically dysphagia and dysphonia (PDD), were evaluated during each follow-up visit using validated questionnaires.

### 2.6. Objective Laryngeal/Phonation Assessment

In cases where patients presented with severe or persistent swallowing and/or voice dysfunction postoperatively, an objective laryngeal assessment was performed using FEES (Flexible Endoscopic Evaluation of Swallowing) by ENT specialists according to standard clinical protocols.

### 2.7. Assessment of Postoperative Dysphagia and Dysphonia

To assess phonation and swallowing complications following anterior cervical spine surgery, two validated instruments were administered: the Ohkuma questionnaire (Figure 3) and the Hospital for Special Surgery Dysphagia and Dysphonia Inventory (HSS-DDI) (Figure 1 and Figure 2). The Ohkuma questionnaire, a 15-item tool, evaluates dysphagia symptoms across all three phases of swallowing. Responses are rated on a Likert scale (Very Often, Sometimes, and Never), scored from 1 to 3, respectively. Patients responding “Very Often” to at least one question were classified as dysphagic while all others were categorized as non-dysphagic. This questionnaire has been translated and validated in Greek and is recognized as a reliable tool for diagnosing and managing dysphagia [20]. The HSS-DDI is a questionnaire specifically developed for the assessment of postoperative PDD following ACDF and comprises 31 items: 20 related to swallowing and 11 to phonation. Responses are recorded on a Likert scale (1—Continuous, 2—Many times, 3—Several times, 4—Few times, and 5—Never), with scores converted to a percentage, where 100% represents normal function without symptoms [21]. Given its specificity for ACDF-related PDD, the HSS-DDI provides a more sensitive evaluation of risk factors for postoperative complications and underwent a two-way cross-cultural adaptation process for use in this study [22]. The Ohkuma questionnaire and the HSS-DDI were administered at 1 week and 1 month postoperatively.

### 2.8. Ethical Considerations

This study was conducted in accordance with the principles of the Declaration of Helsinki. Ethical approval was obtained from the Ethics Council of the University of Ioannina, and all patients provided informed consent before participation. Confidentiality and anonymity of patient data were strictly maintained throughout the study.

### 2.9. Data Analysis

Data analysis was carried out using the Statistical Package for the Social Sciences (SPSS, Windows version) [23]. Two types of statistical analyses were performed: (a) descriptive statistics to summarize patient demographics and clinical characteristics, and (b) inferential statistics for hypothesis testing and comparisons. Continuous variables were presented as means and standard deviations, while categorical variables were expressed as frequencies and percentages. Reliability (internal consistency) of the questionnaires was assessed using Cronbach’s alpha. Comparisons of the total scores of the Ohkuma and HSS-DDI questionnaires between the two time points (one week and one month postoperatively) were performed using the Wilcoxon signed-rank test for paired samples. Analysis of variance (ANOVA) and Tukey’s post hoc test were used to explore differences in questionnaire scores across BMI categories and to identify specific group differences. Statistical significance was set at *p* < 0.05.

## 3. Results

### 3.1. Demographic Characteristics of the Participants

Demographic data of the survey participants were collected as part of the data gathering process (Table 1). The majority of participants were male (60%), while 40% were female. The average age was 54.78 years, ranging from 34 to 74 years. BMI classifications, based on WHO criteria, indicated that 30% of participants were classified as having a normal weight, 47.5% as overweight, and 22.5% as obese. Regarding marital status, 67.5% of participants were married, 10% were unmarried, 10% were divorced, and 12.5% were widowed. In terms of educational background, 37.5% had completed high school, 32.5% had completed university, 12.5% had pursued postgraduate studies, 10% had completed vocational education, and 7.5% had only completed primary school.

### 3.2. Description of Health History

The results of the health history of the participants showed that 87.5% of the participants had a person who cared for them when they returned from the hospital. Regarding the smoking habit, 57.5% responded negatively and 42.5% responded positively. Then 35% of the participants answered that there was a history of psychiatric illness. Of the fourteen who responded positively nine reported that the history was related to depression, four to anxiety disorders and one to schizophrenia. In response to the question whether they had had spinal surgery in the past, 12.5% responded positively. Of these five subjects who answered positively, three had had cervical spine surgery. Finally, 75% answered that they were taking medication. This treatment was mainly related to diabetes, blood pressure regulation, and thyroid (Table 2).

### 3.3. Description of the Disease History

The highest percentage of 32.5% reported symptoms from 7 months to one year before surgery and 27.5% up to 6 months before surgery. Overall, 60% of the participants had surgery within 1 year of the onset of symptoms, 22.5% from 1 year to 5 years, and 17.5% after 5 years. The decision to operate was due in 58% of cases to pain, 18% to a doctor’s advice, 18% to paralysis of the arm, and 8% to dyskinesia. The hernia removed was among A5-A6 in 31 cases, A6-A7 in 27 cases, A4-A5 in 17 cases, A3-A4 in 8 cases, and A7-TH1 in one case.

### 3.4. Swallowing Problems

The survey participants were then asked if they had swallowing problems before surgery. Responses were given on a Likert scale, where 0—Not at all, 1—Almost not at all, 2—Slightly, 3—Very little, 4—Slightly more, 5—Fairly, 6—Much, and 7—Very much, and 65% responded negatively. The mean score of the grade that affected them was equal to 3.13 (SD = 1.68), indicating a small degree of annoyance, and the mean time they experienced these problems was equal to 26 months (SD = 22.21), varying between 6 and 60 months (Table 3).

### 3.5. Phonation Problems

Finally, survey participants were asked if they had any phonation problems before surgery. Responses were given on a Likert scale, where 0—Not at all, 1—Almost not at all, 2—Slightly, 3—Very little, 4—Slightly more, 5—Fairly, 6—Much, and 7—Very much, and 65% responded negatively. The mean score of the grade that affected them was equal to 2.93 (SD = 1.44), indicating a small degree of annoyance, and the mean time that they experienced these problems was equal to 18.29 months (SD = 15.59), varying between one and 60 months (Table 3).

### 3.6. Internal Consistency Assessment of the Ohkuma and HSS-DDI Questionnaires (Cronbach’s Alpha)

The internal consistency of the Ohkuma and HSS-DDI questionnaires was evaluated at two key time points (one week and one month postoperatively) using Cronbach’s alpha coefficient. This analysis is fundamental for establishing the reliability of these tools in measuring dysphagia and dysphonia symptoms in the Greek patient population following ACDF.

#### Cronbach’s Alpha Results

As shown in Table 4, both the HSS-DDI and Ohkuma questionnaires demonstrated high internal consistency at both time points. Cronbach’s alpha values for the HSS-DDI exceeded 0.87, while the Ohkuma questionnaire consistently achieved coefficients above the generally accepted threshold of 0.80.

Values greater than 0.70 are considered adequate for research purposes, above 0.80 are regarded as good, and above 0.90 as excellent. These results confirm that both tools are reliable for assessing dysphagia and dysphonia in patients after ACDF surgery.

The slight decrease in Cronbach’s alpha at the second administration for both tools is a typical observation and may reflect increased homogeneity in patient responses during recovery, as well as reduced variability in symptom severity.

### 3.7. Comparison of Total Scores Using the Wilcoxon Signed-Rank Test

To compare the total scores of the Ohkuma and HSS-DDI questionnaires at two time points (one week and one month after surgery), the non-parametric Wilcoxon signed-rank test for paired samples was applied. The use of the Wilcoxon test is justified due to both the limited variance in the data and the non-normal distribution of the samples, as indicated by normality checks.

#### 3.7.1. Results for the Ohkuma Questionnaire

For a sample size of n = 40 and a significance level of α = 0.001, the critical value for the Wilcoxon signed-rank test is 242. According to the results, the test statistic (26.5) is much lower than 242; so, the null hypothesis is rejected and it is considered that there is a statistically significant difference between the two measurements at the *p* < 0.001 level (Table 5).

The total score of the Ohkuma questionnaire increased significantly from the first to the second measurement (mean from 32.36 to 35.11). The Wilcoxon test statistic exceeded the threshold for statistical significance (*p* < 0.01), confirming a substantial improvement in dysphagia symptoms at one month after surgery. This test is considered particularly rigorous for this sample size due to the low critical value, enhancing the reliability of the finding.

#### 3.7.2. Results for the HSS-DDI Questionnaire

A similar trend was observed for the HSS-DDI questionnaire, where the mean total score increased from 117.27 to 135.02. Here, too, the Wilcoxon signed-rank test revealed a statistically significant difference between the two time points (*p* < 0.01), indicating significant clinical improvements in swallowing and voice-related symptoms during the early postoperative period (Table 6).

#### 3.7.3. Overall Interpretation and Clinical Value

The simultaneous statistically significant increases in the scores for both tools indicate strong and objectively measurable improvements in both dysphagia and dysphonia following ACDF. The use of the Wilcoxon signed-rank test provides increased robustness of these conclusions due to its methodological rigor and its ability to detect differences even in small samples.

### 3.8. Analysis of Variance (ANOVA) of Total HSS-DDI Scores by BMI

In the next stage of the statistical analysis, the body mass index (BMI) was examined as a significant factor influencing the recovery of swallowing and phonation symptoms, as reflected in the total score of the HSS-DDI questionnaire. The analysis was performed for both time points (1 week and 1 month post-surgery) using analysis of variance (ANOVA) among the three BMI categories (normal weight, overweight, and obese).

It was found that there was a statistically significant difference in the total HSS-DDI scores between BMI groups at both the first measurement (*p* < 0.05) and the second measurement (*p* = 0.046). Specifically, patients with a normal BMI consistently had higher mean HSS-DDI scores (i.e., better postoperative function) compared to overweight and obese patients, who exhibited the lowest scores, indicating more persistent dysphagia and dysphonia symptoms after surgery (Table 7).

#### 3.8.1. Application of Tukey’s Post Hoc Test

Following the identification of a statistically significant difference via ANOVA, Tukey’s post hoc test revealed that all pairwise comparisons between the BMI groups were statistically significant at the second measurement (*p* < 0.05):

– Patients with normal weight had on average 19.2 points higher total HSS-DDI scores than obese patients.
– Obese patients had significantly lower scores than overweight patients by 8.5 points.
– Overweight patients also had lower scores compared to normal weight patients (Table 8).

#### 3.8.2. Conclusions and Clinical Significance

This difference highlights that an increased BMI is a critical negative prognostic factor for the postoperative recovery of dysphagia and dysphonia symptoms. The obese patient group presents more persistent and less improved symptoms according to HSS-DDI scores, while normal weight patients demonstrate a better clinical course as early as one month postoperatively.

These findings underscore the importance of a preoperative assessment and management of body weight as a preventive strategy to optimize postoperative outcomes in patients undergoing ACDF.

### 3.9. Assessment of Sampling Adequacy and Limitations of the Factor Analysis

Both the Kaiser–Meyer–Olkin (KMO) measure of sampling adequacy and Bartlett’s test of sphericity were applied to our sample for both questionnaires. The results from these methods consistently indicated that the data were not suitable for a factor analysis. Specifically, the KMO values were below the recommended threshold, suggesting insufficient shared variance among items, while Bartlett’s test, although significant, could not compensate for the low sampling adequacy. Therefore, with the current sample size and data structure, a reliable factor analysis could not be performed for either questionnaire.

## 4. Discussion

The present study provides a comprehensive analysis of postoperative dysphagia and dysphonia in Greek patients after anterior cervical discectomy and fusion (ACDF) using two patient-reported outcome measures: the validated Ohkuma questionnaire and the HSS-DDI, an ACDF-specific instrument for the assessment of both dysphagia and dysphonia. 

### 4.1. Incidence and Severity

Both swallowing and phonation difficulties were frequent pre- and postoperatively, with preoperative symptom severity typically mild to moderate, as reflected by the mean severity scores (on a 0–7 scale). The intensity of these symptoms was assessed with specific ordinal-scaled questions, revealing that most participants reported mild to moderate impairments prior to surgery. Crucially, there were statistically significant improvements in both the severity and frequency of these symptoms within the first month postoperatively, as shown by higher total questionnaire scores and confirmed with the Wilcoxon signed-rank test (*p* < 0.001).

Both questionnaires demonstrated high reliability (Cronbach’s alpha > 0.80) at both time points, supporting the stability and appropriateness of these tools for this patient population.

### 4.2. Role of BMI

One of the most notable findings was the strong and consistent effect of body mass index (BMI) on postoperative recovery. ANOVA and Tukey’s post hoc analysis revealed that patients with a normal BMI achieved significantly better postoperative scores on the HSS-DDI—both at one week and one month—compared to overweight and obese patients. All pairwise comparisons were statistically significant at the second measurement (*p* < 0.05). Patients with obesity had the most persistent symptoms, while those with a normal BMI displayed the most favorable recovery profile.

### 4.3. Other Risk Factors

No statistical associations were found between outcome scores and other demographic or clinical factors, such as age, gender, psychiatric history, presence of a caregiver, and previous cervical surgery.

### 4.4. Factor Analysis Limitations

Confirmatory factor analysis (KMO and Bartlett’s) demonstrated that the sample size was not sufficient for a robust analysis of tool substructure, indicating the need for larger samples to validate subscales and underlying domains for the Greek population.

### 4.5. Clinical Implications

These results underscore the value of a systematic symptom evaluation with patient-centered, validated tools, revealing that while most patients improve functionally after ACDF, an elevated BMI is a pronounced risk factor for delayed or persistent postoperative dysphagia and dysphonia. This highlights the clinical necessity for risk stratification and targeted perioperative management in higher BMI groups.

## 5. Conclusions

This study confirms that dysphagia and dysphonia are frequent but typically improving complications following ACDF. The systematic use of reliable, adapted questionnaires enabled the objective tracking of patient symptoms, capturing a clear trend of postoperative recovery within the first month.

### 5.1. Key Findings

- The majority of patients exhibited significant improvement in swallowing and voice symptoms by the first month postoperatively, regardless of the initial severity.
- An elevated BMI was strongly associated with more persistent and severe symptoms, suggesting that it is a critical modifiable risk factor.
- Both the HSS-DDI and Ohkuma questionnaires were reliable and practical for Greek clinical settings.
- No other demographic or clinical variables showed a significant association with postoperative outcomes.
- Factor analysis efforts were limited by the sample size, pointing to the need for future research with larger cohorts.

### 5.2. Clinical Relevance

Routine preoperative assessment and perioperative management should include risk stratification for patients with a higher BMI to optimize recovery. Further large-scale studies are needed to refine these tools and deepen the understanding of the mechanisms behind persistent dysphagia and dysphonia after ACDF.

## Figures and Tables

**Table 1:** Demographic characteristics.

Variable	Value/Distribution
Mean age	54.8 years (range: 34–74 years)
Gender	24 males (60.0%), 16 females (40.0%)
Mean weight	81.6 kg (range: 51–105)
Mean height	172.8 cm (range: 150–190)
BMI categories	Normal: 12 (30.0%), Overweight: 19 (47.5%),
	Obese: 9 (22.5%)
Marital status	Unmarried: 4 (10.0%), Married: 27 (67.5%), Widowed: 5 (12.5%), Divorced: 4 (10.0%)
Educational level	Primary school: 3 (7.5%), Middle school: 4 (10.0%), High school: 15 (37.5%), University: 13 (32.5%), Postgraduate: 5 (12.5%)

**Table 2:** Clinical history and preoperative data.

Variable	Value/Distribution
Smoking	No: 23 (57.5%), Yes: 17 (42.5%)
Psychiatric history	No: 26 (65.0%), Yes: 14 (35.0%)
Presence of a caregiver	No: 5 (12.5%), Yes: 35 (87.5%)
Medication intake	No: 10 (25.0%), Yes: 30 (75.0%)

**Table 3:** Preoperative swallowing and voice symptoms and analgesic intake after surgery.

Variable	Value/Distribution
Swallowing problems before surgery	Yes: 14, No: 26
Severity of swallowing problems (0–7)	Mean: 3.13 (SD: 1.68)
Voice problems before surgery	Yes: 14, No: 26
Severity of voice problems (0–7)	Mean: 2.93 (SD: 1.44)
Took analgesics after surgery	Yes: 38, No: 2

Severity scores: 0 = no symptoms, 7 = most severe symptoms.

**Table 4:** Internal consistency of the HSS-DDI and Ohkuma questionnaires (Cronbach’s alpha).

Questionnaire	1st Administration (1 Week)	2nd Administration (1 Month)
HSS-DDI	0.92	0.87
OHKUMA	0.85	0.80

**Table 5:** Comparison of total Ohkuma questionnaire scores at 1 and 4 weeks postoperatively (Wilcoxon signed-rank test).

**Measurement**	**Mean Total Score**	
1st Measurement (1 week)	32.36	
2nd Measurement (1 month)	35.11	
**Wilcoxon Statistic**	***p*-Value**	**Conclusion**
26.5 < 242	*p* < 0.01	Statistically significant improvement (*p* < 0.01)

**Table 6:** Comparison of total HSS-DDI questionnaire scores at 1 and 4 weeks postoperatively (Wilcoxon signed-rank test).

**Measurement**	**Mean Total Score**	
1st Measurement (1 week)	117.27	
2nd Measurement (1 month)	135.02	
**Wilcoxon Statistic**	***p*-Value**	**Conclusion**
4.0 < 242	*p* < 0.01	Statistically significant improvement (*p* < 0.01)

**Table 7:** Total HSS-DDI scores by BMI category at one week and one month postoperatively and ANOVA results.

**BMI Category**	**Mean HSS-DDI Score (1st Measurement)**	**Mean HSS-DDI Score (2nd Measurement)**	
Normal Weight	129.4	147.2	
Overweight	118.7	131.0	
Obese	110.2	125.4	
	***p*-Value**	***p*-Value**	**Conclusion**
	*p* < 0.05	*p* < 0.05	Statistically significant difference in HSS-DDI scores by BMI

**Table 8:** Tukey’s post hoc test results for HSS-DDI scores by BMI category.

Comparison	Mean Difference	*p*-Value	Significant Difference
Normal vs. Obese	19.2	*p* < 0.05	Yes
Normal vs. Overweight	10.7	*p* < 0.05	Yes
Obese vs. Overweight	−8.5	*p* < 0.05	Yes

## Data Availability

The data presented in this study are available on request from the corresponding author.
